# Overview of MR Image Segmentation Strategies in Neuromuscular Disorders

**DOI:** 10.3389/fneur.2021.625308

**Published:** 2021-03-25

**Authors:** Augustin C. Ogier, Marc-Adrien Hostin, Marc-Emmanuel Bellemare, David Bendahan

**Affiliations:** ^1^Aix Marseille Univ, Université de Toulon, CNRS, LIS, Marseille, France; ^2^Aix Marseille Univ, CNRS, CRMBM, UMR 7339, Marseille, France

**Keywords:** MRI, neuromuscular disorders, muscle, image segmentation, deep learning

## Abstract

Neuromuscular disorders are rare diseases for which few therapeutic strategies currently exist. Assessment of therapeutic strategies efficiency is limited by the lack of biomarkers sensitive to the slow progression of neuromuscular diseases (NMD). Magnetic resonance imaging (MRI) has emerged as a tool of choice for the development of qualitative scores for the study of NMD. The recent emergence of quantitative MRI has enabled to provide quantitative biomarkers more sensitive to the evaluation of pathological changes in muscle tissue. However, in order to extract these biomarkers from specific regions of interest, muscle segmentation is mandatory. The time-consuming aspect of manual segmentation has limited the evaluation of these biomarkers on large cohorts. In recent years, several methods have been proposed to make the segmentation step automatic or semi-automatic. The purpose of this study was to review these methods and discuss their reliability, reproducibility, and limitations in the context of NMD. A particular attention has been paid to recent deep learning methods, as they have emerged as an effective method of image segmentation in many other clinical contexts.

## 1. Introduction

Neuromuscular pathologies are rare diseases that can occur in both children and adults. Very few therapeutic strategies have been proposed so far. Reliable outcome measures that could be sensitive enough to detect therapeutic effects are still missing. Diagnosis of neuromuscular pathologies is commonly based on clinical presentation, genetic testing, and histological assessment of muscle biopsy. Given its non-invasiveness and its ability to distinguish fat and muscle tissue, magnetic resonance imaging (MRI), and more particularly quantitative MRI (qMRI), has emerged in recent years as a tool of choice for the investigation of neuromuscular diseases (1). Over the last 20 years, research projects have been developed to define relevant and sensitive MRI parameters that could be used in the diagnostic classification and the follow-up of neuromuscular diseases (2–6). The initial approaches were based on a visual analysis of hypersignals illustrating pathological processes and on that basis alterations patterns have been proposed. In slowly progressive diseases, such as neuromuscular disorders, the sensitivity of such visual qualitative assessments is largely questionable and may not be powerful enough to identify mild changes in muscle function that occur from year to year.

More recently, qMRI have been used in order to generate parametric maps illustrating the various pathological processes occurring in skeletal muscle, i.e., mainly inflammation and fat infiltration (4, 5). Compared to visual scores, such a quantitative approach has paved the way of a more sensitive assessment of dystrophies and neuropathies. Beyond the diagnostic interest, these approaches provide sensitive and reproducible biomarkers, which have been used for follow-up studies (6–8). In addition to the generation of parametric maps, qMRI has to be combined to images segmentation if one intends to extract the relevant information within different regions of interest. Segmentation refers to the delineation of muscle regions of interest that must be distinguished from subcutaneous and perimuscular adipose tissues, on the one hand, and from bones, on the other hand. Segmentation in general and segmentation of MR images in particular is a time-consuming process so that automatic procedures are highly requested. However, automation of muscle segmentation in MR images is very challenging given the poor contrast between different muscles and the large variability of muscle shapes (9). In pathological situations, the challenge can be even higher given that borders between the different compartments can be hidden by a severe fat infiltration. Given the task complexity, most of the studies related to the investigation of neuromuscular diseases have been based on the manual segmentation of individual muscles or muscle compartments. One has to keep in mind that such an approach is operator dependent and time consuming. As a result, quantitative analyses have been mainly performed over a limited number of slices and not on the whole 3D datasets (4, 10) or on a limited number of individual muscles (6). In a few clinical studies in which the manual segmentation has been performed in the 3D field of view, an inconsistent distribution of MRI biomarker values has been interestingly reported along the proximo-distal axis (2, 11–13). In addition, it has also been documented that individual muscles could be affected differently and that this difference could also occur among patients and neuromuscular disorders (5, 14–16). These results clearly emphasized the need of reliable automatic 3D segmentation methods and the relevance of evaluating muscles individually, rather than by muscle groups.

Over the last 15 years, several automated methods have been reported in the literature with the aim of segmenting muscle groups or individual muscles in MR images. Although promising, most of these methods have been tested in MR images from healthy volunteers for which fat infiltration and atrophy were absent so that the corresponding confounding factors could not be taken into account. More recently, a few automatic methods have addressed the issue of segmenting MR images of patients with neuromuscular disorders but only for the delineation of muscle compartment.

Very recently, semi-automatic methods have been reported for individual muscles segmentations in order to reach an optimized balance between segmentation accuracy and user's dedication. These full 3D methods have been successfully used in a clinical context. A few limitations has to be acknowledged for these kinds of methods. They are still time consuming and require a manual initialization so that reliable full automatic segmentation methods are still warranted for individual muscles.

Deep learning methods have been very scarcely used in the field of neuromuscular disorders and considering the results obtained in other scientific fields, they might represent a very interesting alternative for a full-3D segmentation of MR images. One should keep in mind that large databases should be available for this kind of approach and this could be a limitation in rare diseases.

Manual segmentation methods are not applicable for 3D datasets and the follow-up of neuromuscular diseases. They have been recognized as time consuming (5 h per subject for the 3D manual segmentation of 4 muscles) and operator dependent (3.1% volume error for the *quadriceps femoris* in healthy subjects) (9). On that basis, these methods were beyond the scope of the present review. Considering that neuromuscular disorders have been mainly studied using MRI of thighs and legs, only the automated methods that have been used for the segmentation of lower limbs images are part of the scope of the present review.

The main aim of this review is to provide an overview of the methods dedicated to the segmentation of individual skeletal muscles on MR images and to discuss their validity and reliability. We pay a particular attention to the evolution of segmentation strategies, from the separation of muscle and fat deposits to the segmentation of individual muscles, together with the clinical potential and applicability in the context of neuromuscular disorders. Finally, we introduce insights into semi-automatic methods that could potentially break the barrier between research and clinics. These methods could provide clinicians with user-friendly tools that generate biomarkers for individual muscles over an entire 3D dataset. The emerging segmentation methods based on deep learning approaches have been included in a dedicated section as they are still emerging.

## 2. Muscle Tissue Segmentation Issues

### 2.1. Type of MR Images for Segmentation

Since the emergence of MRI, the quality and type of images available through this acquisition modality have greatly evolved and have consequently influenced automated segmentation methods. First segmentation methods had to deal with severe artifacts on MR images and hence segmentation of the contour of lower limbs and muscular region were a complicated task (17). Over the years, image quality has been dramatically improved with hardware and images techniques advancement. Intensity inhomogeneity correction method, such as N3 and more recently N4 algorithm (18), also strongly contributed to the improvement of image quality and such algorithms are now a common pre-processing step for muscle segmentation method. Segmentation methods for lower limbs MR images were first dedicated to T_1_-weighted images, commonly used in clinics. The parametric images from qMRI sequences were then used as they may display different information regarding the nature of tissue (**Figure 3**). In most of the studies discussed in this review, parametric maps used for segmentation methods were extracted from multi-echo chemical shift-based water-fat separation MR sequences (19).

### 2.2. Regions of Interest

**Figure 1:**
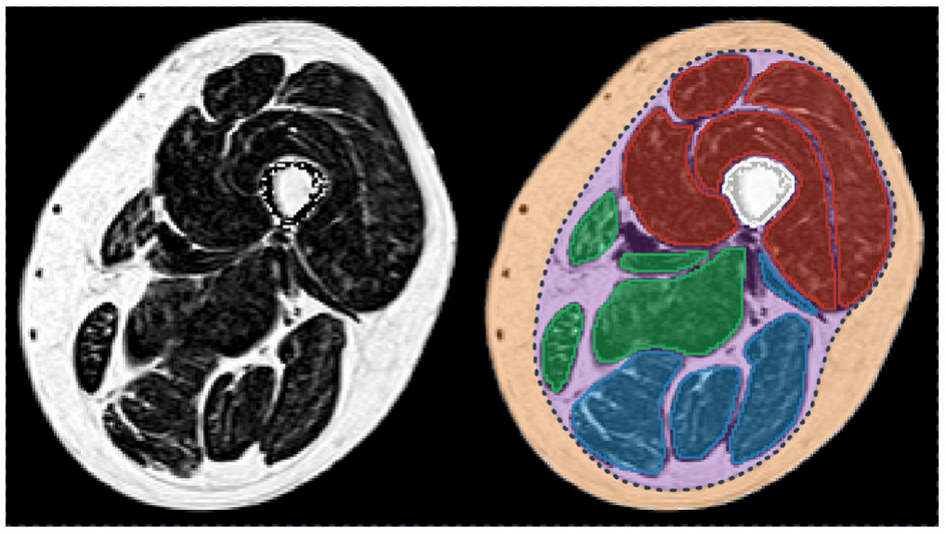
Fat fraction map of a thigh of a patient with myotonic dystrophy type 1 **(left)** and corresponding segmentation of the principal regions of interest **(right)**. Femur is in white. The individual muscles of the knee extensors, knee flexors, and the medial compartment are in red, blue, and green, respectively. Subcutaneous (orange) and perimuscular (purple) tissues are separated by the *fascia lata* (dot line in dark blue). Patient participated in the randomized controlled trial OPTIMISTIC (5).

As illustrated in Figure 1, different tissues are visible in a MR image of a lower limb. For the sake of clarity, these tissues will be designated according to the nomenclature of (20). The subcutaneous adipose tissue (SAT) and the internal adipose tissue (IAT) are separated by the *fascia lata* for the thigh and by several deep fascias for the lower leg. Within the IAT, the intramuscular adipose tissue is defined as the adipose tissue contained within muscles while perimuscular adipose tissues (PAT) designates the remaining adipose tissue, mainly the fat deposits between the muscles.

**Figure 2:**
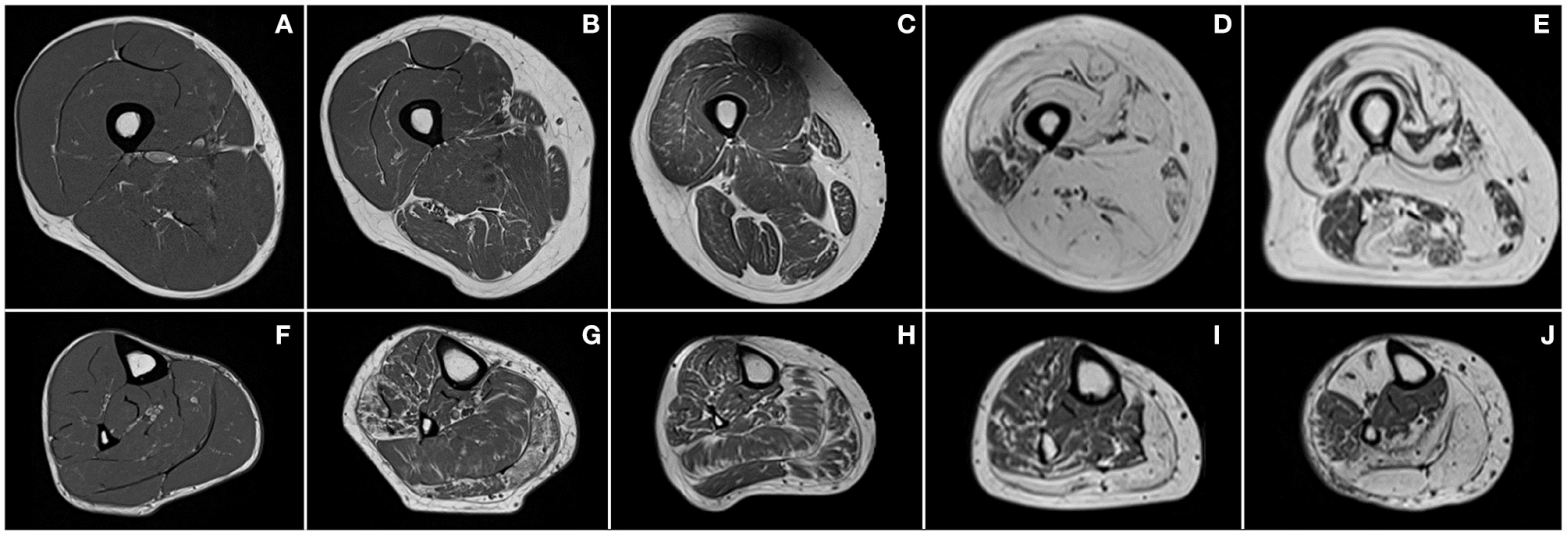
Examples of T_1_-weighted images of thighs (first row) and lower legs (second row) of a healthy subject **(A,F)** and patients with Charcot–Marie–Tooth disease type 1A **(B,G)**, myotonic dystrophy type 1 **(C,H)**, facioscapulohumeral muscular dystrophy **(D,I)**, and inclusion body myositis **(E,J)**.

In healthy subjects, fat is mainly present as SAT, whereas IAT is almost absent (Figure 2A). On the contrary, in neuromuscular diseases, muscle tissue is submitted to histological changes leading to a progressive replacement of muscle tissue by adipocytes. Intramuscular fat infiltration can even lead to muscle necrosis and fibrosis (Figure 2D).

Segmentation strategies have evolved over the years with improvements in image quality and clinical needs. The first approaches intended to separate muscle and fat deposits with no distinction between subcutaneous and internal compartment fat. Clinical research has revealed that perimuscular and intramuscular adipose tissue are not part of the same metabolic processes (21) but their respective contributions are not yet fully understood (22). Segmentation strategies have therefore evolved toward the segmentation of muscle regions or individual muscles in order to allow the precise quantification of each adipose compartments. This has also been facilitated by improved image quality, which has allowed better visualization of the boundaries between tissues. It should be noted that the nomenclature of the different adipose tissues is still not clearly defined in the research field of segmentation strategies. The terms “intermuscular fat” and “intramuscular fat” have been confused and misleadingly used in many of the articles we reviewed in this study.

### 2.3. Validation of Segmentation Approaches

Validation is a crucial step in the development of automatic segmentation methods. It intends to assess the effectiveness of an automatic segmentation method based on the comparative analysis between the automatic segmentation provided by an algorithm and a ground truth produced by one or more experts in the field, usually radiologists.

Several complementary metrics have been commonly used. They can assess the overlap between segmentations, the distance between segmentation contour points or the volumes computed from the segmentations. Each metric is actually sensitive to one type of segmentation error (size, location, and shape) and none can take all error types into account (23).

For the muscle segmentation methods reported in the present review, the most commonly used metrics are the relative volume difference (RVD), the dice similarity coefficient (DSC), the Hausdorff distance (HD), and the average surface distance (ASD).

Let *X* be the segmentation resulting from the algorithm and *Y* the ground truth. RVD is computed taking into account the volumes quantified from the manual and automatic segmentations. It actually refers to the ratio between the |*X* − *Y*| difference and |*Y*|. The RVD score is a relevant metric in clinical fields related to muscle because many studies used muscle volume change as a biomarker. Nevertheless, this metric does not allow a geometric and spatial analysis between the manual and automatic segmentations.

The DSC measures the relative overlap between *X* and *Y*. It is calculated as the ratio of twice the intersection between *X* and *Y* by the number of combined elements of *X* and *Y*. As defined in (1), DSC can also be expressed in terms of true positive (TP), false negative (FN), and false positive (FP). The DSC values can range from 0 to 1, 1 indicating the largest similarity between segmentations.

(1) $$\begin{array}{l} DSC=\frac{2|X\cap Y|}{\left|X|+|Y\right|}=\frac{2\cdot \text{TP}}{\text{FN}+2\cdot \text{TP}+\text{FP}} \end{array}$$

In addition to DSC, the distance between the segmentation boundaries can be computed in order to assess the segmentation robustness regardless of the volume. Let ∂*X* be the segmentation boundary of *X* and ∂*Y* be the boundary of *Y*. For HD and ASD, the smallest distance separating the boundaries is measured between each point of ∂*X* and ∂*Y*. The distance between two points *x* and *y* is the Euclidian distance δ(*x, y*) = ||*x* − *y*||. The HD is calculated as the maximum of these distances (2) and the ASD as the average of the distances (3). Both distances are expressed in millimeters and low values are desirable for an accurate segmentation.

(2) $$\begin{array}{l} \text{HD}\left(\partial X,\partial Y\right)=\max\left\{\underset{y\in \partial Y}{\sup}\underset{x\in \partial X}{\inf}\delta \left(x,y\right),\underset{x\in \partial X}{\sup}\underset{y\in \partial Y}{\inf}\delta \left(x,y\right)\right\} \end{array}$$

(3) $$\begin{array}{l} \text{ASD}\left(\partial X,\partial Y\right)=\frac{\sum_{x\in \partial X}\delta \left(x,\partial Y\right)+\sum_{y\in \partial Y}\delta \left(\partial X,y\right)}{\left|\partial X|+|\partial Y\right|} \end{array}$$

The three geometric metrics, DSC, HD, and ASD, are complementary and should all be evaluated in order to properly validate a segmentation method. The DSC and ASD provide global information on the segmentation, i.e., the overlap and the distance between the boundaries, respectively. Unlike DSC and ASD, HD is very sensitive to outliers and to slight shape differences.

## 3. Evolution of Segmentation Strategies

### 3.1. Automatic Separation Between Muscle and Fat Deposits

Over the last 15 years, several automated methods have been reported in the literature with the aim of separating muscle and fat tissue in lower limbs MR images.

#### 3.1.1. Separation Between Muscle and Adipose Tissue

Early automated segmentation methods took advantage of the contrast between fat, muscle, and bone tissues. Accordingly, Mattei et al. proposed a method for the semi-automatic segmentation of the muscle compartment of the thigh based on a histogram representation of T_1_-weighted images (24). Based on user-defined thresholds for muscle and fat pixel intensities, the method was validated through a reproducibility study of the results between 3 experts but no metric related to the segmentation accuracy was reported. Becker et al. proposed an automatic method for the separation between muscle and fat using a series of thresholding, morphological, and connectivity operations enhanced by the use of the four different kind of images provided by the chemical-shift DIXON sequences (25). They reported a DSC score larger than 0.95 for muscle segmentation performed in seven slices selected along the lower limbs of four subjects.

Threshold-based methods are fast and simple to implement but are quite sensitive to noise, imaging artifacts, and above all need empirical thresholding. Automatic methods such as K-means clustering have been developed to address this issue (26). These methods classify tissues according to the intensity of each pixel. Other automatic methods based on Gaussian mixture model (GMM) histogram analysis have been reported (27). The corresponding results were better because of the unsupervised learning, which allows the algorithm to adapt to each image and makes it more robust.

Because K-means clustering approaches are based on the assumption that a feature vector belongs to only one class, they have been recognized as ill-suited for MR image segmentation when classes overlap or when the information is unclear and uncertain (28). Partial volume effects between muscle and fat near muscle boundaries and inter- or intra-muscular fat infiltration lead to class uncertainties. Fuzzy c-means (FCM) clustering algorithm has been developed in order to overcome this issue. An FCM clustering algorithm was proposed by Barra et al. to estimate 3D volumes of muscle and fat on thigh images (29). The method was reproducible with respect to volume estimates in five images acquired on the same day from three subjects but no comparison was performed with ground truth segmentations.

Methods based on clustering or histogram analysis allowed fat and muscle tissues to be distinguished but SAT and IAT remained undistinguishable. This is of importance considering that IAT is directly related to the pathological process of neuromuscular disorders, whereas SAT is not.

#### 3.1.2. Separation Between Perimuscular and Subcutaneous Adipose Tissue

Since subcutaneous and internal adipose tissue have to be distinguished, several approaches have been proposed for the segmentation of the internal SAT border, in addition to fat and muscle tissue separations.

Although Valentinitsch et al. (30) applied K-means clustering on the different images resulting from chemical shift-based water-fat separation MR sequences successively, Yang et al. (31) proposed a machine learning algorithm using the whole set of images at a time. Both of these approaches allowed IAT to be distinguished from SAT using basic morphological operations such as dilatation, erosion, and connected components. These methods were assessed on a single chosen slice position for Valentinish et al. and on a 3D stack of slices of thigh for Yang et al. For both methods, good results were obtained for the segmentation of muscular tissue in “healthy” images with DSC values higher than 0.94 and ASD around 0.80 mm. However, Valentinish et al. highlighted that this kind of approaches may not identify the correct muscle envelope if a muscle next to the SAT region is fatty infiltrated or surrounded by a substantial amount of fat, a common scenario in patients with neuromuscular disorders (Figure 2). Moreover, defining the delimitation between IAT and SAT as the muscular envelope was misleading given that the true natural boundary is the *fascia lata* (20), which may not appear close to the muscle.

To address the issue of segmenting the *fascia lata*, which is a very thin tissue, poorly contrasted, and partially invisible in MR images, a few methods based on snake, active geodesic contours (32) have been proposed.

This kind of algorithm may be able to perform an active contour evaluation toward weak edges, such as that of *fascia lata* (Figure 1). Snake algorithm was used in similar methods on T_1_-weighted images by (33, 34) with a difference regarding contour initialization. Although Makrogiannis et al. used the leg boundary segmented by morphological operations, Orgiu et al. implemented a rough *fascia lata* segmentation defined by the muscle envelope segmented with an FCM clustering followed by morphological operations. Positano et al. used a gradient vector flow snakes (35), an extension of snake active contours, which does not need to be initialized close to the boundary and is able to converge to the boundary concavities (27). Succession of active contours initialized by a circle surrounding the leg were applied to segment the external SAT contour, the internal SAT contour, and finally the bone contour. External force of the snakes used edge map derived of a fat map previously created with an FCM clustering. Inside the internal border of the SAT, Positano et al. used a GMM approach to separate fat and muscle in the perimuscular region, whereas Makrogiannis et al. applied K-means clustering to the combined space of fat image and water image. These approaches were assessed in MR images of healthy volunteers (33) and obese patients (27, 34). No direct comparison was made with ground truth segmentation but good correlations for volume quantification between manual and automatic segmentations were reported for muscle and fat tissues within the internal SAT border. Orgiu et al. reported a mean ASD value of 0.81 mm for the *fascia lata* segmentation but no indication about the HD.

More recently, several approaches have been proposed to enhance active contour methods for *fascia lata* segmentation with learning methods and line detection filters.

A random forest approach coupled with sparsity constraints to fix the noise caused by veins was proposed by Tan et al. with the aim of learning a 2D *fascia lata* detector and incorporating it into the external energy terms of a gradient vector flow snake (36). They reported a high average DSC of 0.97 and an average ASD of 1.37 mm in thigh images of osteoarthritis patients. This method clearly outperformed those based on classic active contour model (37). In two others similar methods, geodesic active contours, also initialized with muscle envelope, were enhanced by line detection filter, which extracts *fascia lata* point candidates (38) and a vessel enhancement filtering, which distinguishes plate-like shapes (39). Kovacs et al. assayed their method on T_1_-weighted images of myopathic patients and DSC for the segmentation of non-affected and mildly affected muscles was 0.93, whereas it was reduced (0.80) for severely affected muscles. These results can be explained by the volume dependency of the DSC and the fact that muscle volume was lower in severely affected patients. For the detection of the true muscle envelope (i.e., the *fascia lata*), HD scores were systematically high with an average of 13 mm regardless of the pathology severity. Chaudry et al. proposed a semi-automatic method based on live-wire to refine the *fascia lata* automatic delineation in addition of their automatic approach. Manual corrections were partially necessary for 40% of the datasets (23 healthy young men and 50 elderly sarcopenic men with a moderate level of fat deposits). No direct comparison with ground truth segmentations was performed.

Although the detection of the muscle envelope with intensity-based clustering or active contour approaches is ill-suited for images of patients with neuromuscular disorders, these methods have been used as an initialization step in most of the studies dedicated to *fascia lata* detection. As expected, segmentation of images with a severe fat infiltration was of poor quality. Other approaches therefore had to be proposed.

Chambers et al. (40) introduced a method based on a live-wire approach for muscle region segmentation. A fingerprint-based algorithm was used to overcome the limitations of basic live-wire approaches, which are sensitive to the additive noise and small textural information. The muscle region border was then identified using an exponential cost function related to the edge information. The internal border of the SAT was first detected by keeping the edges closest to the border of the leg. The procedure was performed on the slice with the largest cross-sectional area and then the border search was restricted to adjacent slices, assuming the location of the boundaries is fairly similar from one slice to another. The method was validated on 10 T_1_-weighted images of facioscapulohumeral muscular dystrophy (FSHD) patients and the DSC and ASD values were 0.89 and 0.10 mm, respectively. They also demonstrated that the state-of-art FCM clustering and active contour methods were less robust than their method when fat infiltration was present.

Very recently, Gadermayr et al. (41) evaluated up-to-date approaches for the segmentation of the whole muscle region in datasets of patients with neuromuscular disorders. For mildly to moderately infiltrated patients, all the tested approaches allowed an accurate segmentation with mean DSC values above 0.90. For images with a severe fat infiltration, they demonstrated that a graph cut approach incorporating shape knowledge exhibited a more accurate segmentation than the other methods with an average DSC value of 0.80.

#### 3.1.3. Toward Fat Infiltration Measurement

Overall, several studies have been proposed for the segmentation of muscle and internal adipose tissue in MR images of lower limbs. Only a few have been validated for images of patients with neuromuscular disorders with a rather limited number of images and metrics related to segmentation. One has to keep in mind that the initial clinical driving force related to these segmentation methods was related to the automatic quantification of fat-unaffected muscle volume. Accordingly, Müller et al. used intensity-based segmentation approaches on T_1_-weighted images in order to quantify the remaining muscular tissue. The corresponding metric allowed to distinguish myopathic and neuropathic patients from control subjects (42). Similar methods have been used for the estimation of fat infiltration in images of patients with neuromuscular disorders. This clinical purpose faced several issues. Lareau-Trudel et al. reported, in a clinical study, that separation between SAT and IAT with active contour methods failed for patients with severe fat infiltration and manual corrections had to be performed in 20% of the cohort (15). Furthermore, in many of the studies reviewed, authors claimed to propose a quantification of the intermuscular fat fraction while using ratios between fat-unaffected muscle volume and adipose tissue volume contained within the *fascia lata*. No separation between intermuscular and intramuscular fats was performed and could be hardly expected with such intensity-based approaches. The assessment of intramuscular adipose tissue is of utmost importance in neuromuscular diseases given that it is a hallmark of the disease process.

Overall, the segmentation approaches reviewed above are not adequate for the automatic quantification of fat infiltration, whereas automatic segmentation of individual muscles is warranted if one intends to thoroughly assess a pathological process.

### 3.2. Automatic Segmentation of Muscle Regions

The segmentation of individual muscles would have two main advantages. Fat infiltration and other pathological changes could be assessed in individual muscles. In addition, the processing of 3D datasets could be useful to investigate potential changes along the proximo-distal axis. Accordingly, fat infiltration patterns of individual muscles have been reported in a few clinical studies (5, 16).

**Figure 3:**
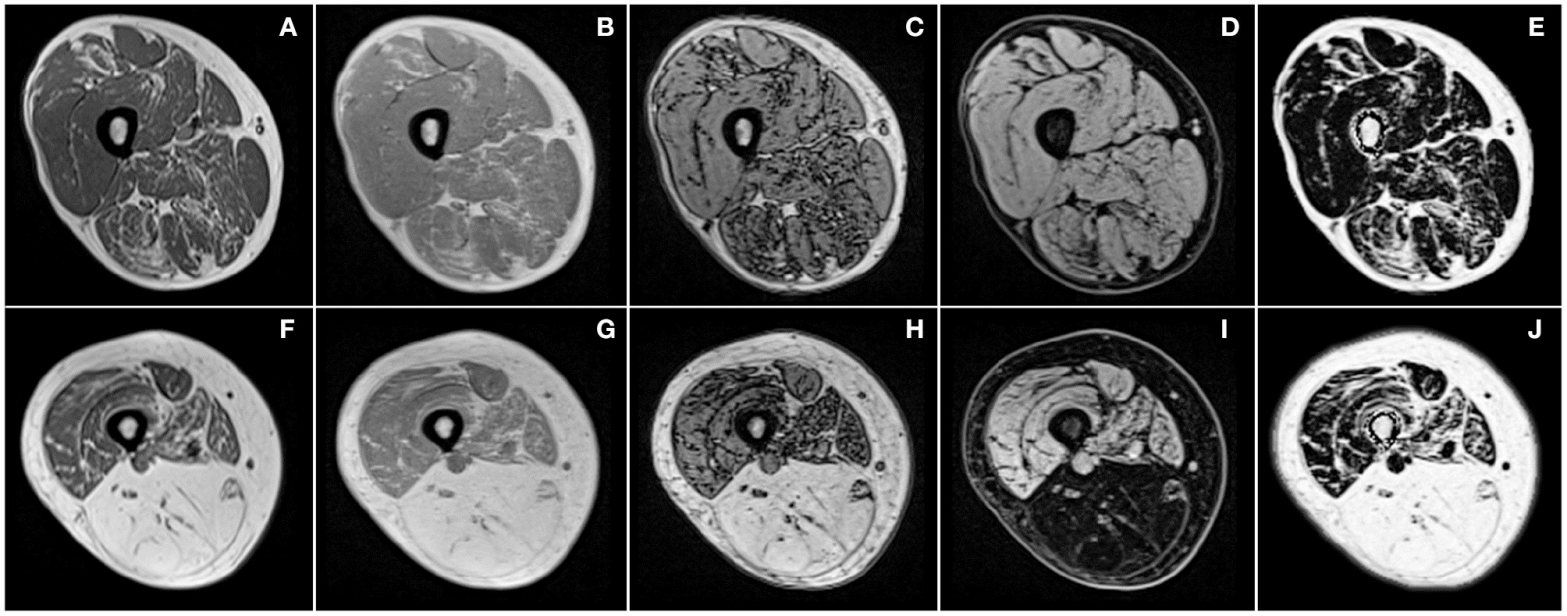
Examples of T_1_-weighted images **(A,F)** and images produced by chemical shift-based water-fat separation MR sequences for the thigh of two patients (first and second row) with different severity of facioscapulohumeral muscular dystrophy. Illustrations correspond to in-phase **(B,G)**, out-of-phase **(C,H)**, water **(D,I)** images, and quantitative fat fraction map **(E,J)**.

Both the fat infiltration severity together with the time-dependent progression can largely vary between muscles in a given patient, or between patients with a given disease (Figure 3) and between various muscle disorders (Figure 2). On that basis, quantification of any MRI biomarker of interest in individual muscles and in a 3D dataset is of crucial importance so that cross-sectional as well as longitudinal studies and therapeutic trials could be properly performed. As most of the coming therapeutic strategies are more likely to halt or slow the disease progression rather than reversing the already established tissue damage, it may be worthwhile to exclude the observation of fully infiltrated muscles in order to pay a more particular attention to those muscles not fully infiltrated.

In addition, automated methods are necessary to study large cohorts of patients with neuromuscular diseases given that manual segmentation is not conceivable given the time required (9).

As mentioned above, the automated segmentation of individual muscles in MR images is challenging for multiple reasons. Muscles in an MR image of healthy subjects display similar intensities and textures so that they can be hardly distinguished. In addition, the boundaries between muscles are very thin and may be discontinuous or invisible in MR images. As illustrated in Figure 2, muscle boundaries can be even less visible in case of fat infiltration. Different muscle shapes and textures within and between patients also contribute to the challenge the automatic segmentation task. In addition, considering that muscles are made up of soft tissues, one can expect large shape changes due to external constraints imposed by leg and/or coil positioning in the MR scanner.

Over the last years, several studies have proposed automated approaches to overcome these difficulties and to provide accurate segmentations of muscles individually or grouped by regions. Most of the methods were based on shape-based approaches given that intensity-based approaches cannot distinguish individual muscles.

A wavelet-based encoding method was proposed by Essafi et al. (43) in order to provide a hierarchical encoding of shape variability. This approach was assessed for the segmentation of the *gastrocnemius medialis* in 20 healthy subjects and five patients. The corresponding results were of poor quality with an averaged DSC of 0.55.

Baudin et al. evaluated a method based on random walk in order to address the issue of incomplete contours, which may occur between muscles. Such a method relies on seeds positioning for the initiation step that can be done manually or automatically using atlases. This method has been evaluated in out-of-phase images, which have the particularity of showing strong contours between tissues and thus between individual muscles in images of healthy subjects. The corresponding DSC values were high, i.e., 0.80 ± 0.19 in 15 control subjects (44). The incorporation of shape prior knowledge and confidence map of muscle contours led to larger DSC values [0.86 ± 0.07; (45)]. Although such a method has not been assayed in patients with neuromuscular disorders, one could expect much lower DSC values given that the fat infiltration should erase the muscle borders. Andrews et al. (46) addressed this issue by designing a random forest boundary detector that seek to learn common appearances of the interfaces between muscles in order to distinguish them from intramuscular fat. This intermuscular boundary detector was combined with a statistical shape model over the space of generalized log-ratio representations and a pre-alignment approach based on GMM segmentation of the muscular tissue. They evaluated their method for the segmentation of individual thigh muscles in 10 healthy subjects and 10 patients with chronic obstructive pulmonary disease and they reported DSC values ranging from 0.70 ± 0.16 to 0.93 ± 0.06.

A method based on active contour model with an initialization through an active shape model was assessed for the segmentation of muscle regions in thigh of patients with knee osteoarthritis (47). The active shape model was trained in 113 axial MR slices for an assessment on 20 images. They reported that 50 training datasets were enough to obtain accurate segmentation. Good DSC values were reported for the segmentation of each muscle region. However, their method was only designed for the segmentation of a unique mid-slice. In addition, a manual interaction of 3–5 min per slice was required for refining the initialization steps of both the active shape model and the active contour model.

#### 3.2.1. Atlas-Based Approaches

Atlas-based approaches have been proposed for the automatic segmentation of individual muscles as they can incorporate spatial prior anatomical knowledge at individual muscles level. Atlas-based segmentation is a well-established concept (48) that has been widely applied to brain structures. These approaches treat segmentation as an image registration problem that aims at computing the optimal transformation fields from the pre-labeled atlases to the new image to be segmented. The atlas labels, once transferred to the new image domain, are merged and result in the final unique segmentation.

Such an approach has been used for the quantitative assessment of regional muscle volume in whole-body MR images (49). A multi-scaled and phase-based morphon method was selected for the registration because it would be less sensitive to MRI inhomogeneities. Water images were used since they display the least anatomical variation and contain the largest amount of information regarding muscle shapes. The multi-atlas process was performed twice, once with all the images of the atlas to obtain a first coarse segmentation, and then a second time using only the atlases having similar volumes to the coarse segmentations. Labels from atlases were merged into a probabilistic map and a threshold was set empirically to produce the final segmentation. Then, muscle and fat volumes were separated inside the segmented areas with a threshold. Using a leave-one-out strategy in 20 healthy subjects, good correlation was reported for muscle volume quantification. The mean TPVF was 93% for lower leg, posterior and anterior thigh compartments, and mean FPVF was 5% for lower leg and 8% for posterior and anterior thigh compartments. No geometric metrics related to the muscle envelope segmentation by multi-atlas process has been reported. In a recent study, this multi-atlas approach was also assessed as sensitive enough to detect significant changes in muscle volume following training activities (50).

Multi-atlas approaches are strongly dependent on the registration model used. Le Troter et al. (51) evaluated different registration methods from well-known open-source libraries to segment the four muscles of the *quadriceps femoris*. They demonstrated that multi-atlas process could be improved with initial registrations guided by the segmentation of SAT, muscle envelope, and bone, using a method described by Positano et al. (27). Similarly to Karlsson et al., the results for the segmentation of the whole *quadriceps femoris* in 25 healthy subjects were good with an averaged DSC score of 0.94 ± 0.03. The results were lower for the *vastus lateralis* and *rectus femoris* muscles, i.e., mean DSC values of 0.88 ± 0.08 and 0.84 ± 0.12. In addition, a 20% volume error with RVD scores of 0.17 ± 0.18 and 0.21 ± 0.24 were reported.

A multi-atlas approach based on B-spline nonrigid registrations has been reported by Belzunce et al. (52) with the aim of segmenting the gluteal muscles on hip and thigh images. Registrations were performed on muscle envelope pre-segmented with the Otsu algorithm. The approach was evaluated on both multi-atlas of T_1_-weighted and DIXON in-phase images of 15 healthy subjects with DSC values of 0.94 ± 0.01 and RVD values of 1.5 ± 4.3%. The assessment was only performed on the medial slices of the dataset excluding the extremities deemed to be more difficult to segment because of more variability and uncertainty.

Very recently, Mesbah et al. (53) introduced a Markov random field model combining appearance and spatial models with the prior shape information from atlases and so in order to segment the three main muscle groups of the thigh. They reported good DSC scores (0.89 ± 0.05 to 0.95 ± 0.03) but the HD scores were of poor quality with an average ranging from 10.51 ± 6.37 to 31.53 ± 14.24 mm for the medial compartment. Furthermore, their method was assayed on images of healthy subjects and patients with spinal cord injury, for whom no fat infiltration occurs. Given that part of their method relied on intensity-based approach, it may be ill-suited for images of neuromuscular disorder patients. However, they demonstrated that their approach may outperformed those based on majority vote or STAPLE fusion following nonlinear atlas-based registrations.

The main advantage of multi-atlas methods is that any tissue compartment can be segmented according to a given atlas. This is of great interest for the study of neuromuscular diseases in which individual muscles are affected differently depending on the pathology. However, multi-atlas approaches have proposed mixed results for the segmentation of individual muscles and have only been validated in images of healthy subjects. In all the methods presented above, the final step of label fusion used only parts of the atlases, i.e., those closest to the image to be segmented. Various merging strategies have been used, i.e., majority vote (52) or more thoughtful algorithms like STEPS (51) or a Markov random field model (53). It should be kept in mind that parameters chosen empirically are optimized for a given training database and could have to be retuned for other images. On that basis, it may be necessary to have access to different subgroups of atlases that may be adapted to different types of images. This feature could be problematic for the generalization of these methods for the segmentation of pathological images considering the large between-muscles and between-subjects phenotypic variability. As neuromuscular diseases are classified as rare diseases, there is currently no database large enough to cover all the variability of pathologies as it may be available for the brain (54).

The above-reviewed approaches were designed for cross-sectional studies and a follow-up version has been originally reported by Le Troter et al. In a so-called single-atlas approach, they used the manual segmentation of a first time point as an atlas for the following time points (51). Since successive MR images of the same subject may show little anatomical variations, single atlas-based nonlinear registration can correctly transfer segmentation of the first time point images to the others. Assessed in healthy subject images, they reported much better results than the multi-atlas based approaches. DSC scores were above 0.89 for each individual muscle of the *quadriceps femoris* with RVD scores below 5%. A limitation of this method is that the full automatic aspect is lost with the need of an initial manually segmentation.

#### 3.2.2. Toward Intramuscular Fat Infiltration Measurement

Although several automatic approaches have reported promising results for the segmentation of muscle regions in healthy subjects, none of them have been assessed for images of patients with neuromuscular disorders. To the best of our knowledge, only a single study has been devoted to the segmentation of muscles in patients with chronic obstructive pulmonary disease for whom fat infiltration was moderate and the approach was not accurate enough to be considered for clinical applications.

Overall, reliable automatic segmentation methods are still warranted for individual muscles in the context of neuromuscular pathologies. This is supportive of the conclusion from the review by Pons et al. (55). Although promising, the results already obtained in images of healthy subjects do not guarantee similar results on images of patients for whom factors such as fat infiltration may be problematic. Another critical issue is the availability of pre-labeled data sets of muscle MR images. The validation of a method requires manual segmentations from experts to be considered as the ground truth segmentations. As we previously mentioned, manual segmentation is time consuming and validation of methods has often been performed using a single slice (47).

The lack of automated segmentation methods is likely the reason why only a limited number of clinical studies have assessed qMRI scores at the individual muscles level in 3D datasets. Most of the studies have been performed considering a few slices only (4, 10) or a limited number of individual muscles (6).

### 3.3. Semi-Automatic Segmentation of Muscle Regions

As no automatic method has been validated for accurate segmentation of individual muscles in neuromuscular disorders, semi-automatic methods have been proposed to reach an optimized balance between segmentation accuracy and user's dedication.

A semi-automatic method for segmenting the whole *quadriceps femoris* was proposed with the manual delineation of a line separating this muscle group from the rest of the muscular envelope automatically pre-segmented with an adaptive threshold on T_1_-weighted images (56). The method was assessed in healthy and elderly subjects and managed to reach a time saving of 87% with a mean DSC of 0.98. As we previously indicated, this kind of intensity-based methods is ill-suited for images of patients with neuromuscular disorders.

Two similar methods have been proposed to generate 3D muscle segmentations from manual segmentation on a limited number of 2D slices. One of the advantages of these approaches is that any tissue compartment can be segmented according to the manual segmentation defined on the few axial slices used as initialization of the process.

Jolivet et al. (57) proposed a model based on parametric-specific object method. Only a fast rough contouring of the muscle using polygons was necessary on the initial axial slices. Polygons were then matched to the muscle shape using an automatic contour optimization based on local gradient weighted by intensity similarity and distance to the rough contouring. Once segmentations were well-tuned on the initial slices, parametric-specific object was constructed and deformed to match the manual muscle segmentation. Process was iterative with the successive injection of the axial interpolated segmentation mask, after automatic contour optimization, in the parametric-specific object. The manual segmentation of only 5 axial slices took 21 min (against 80 min for manual segmentation of all slices) and was enough to obtain an accurate 3D segmentation of all individual muscles with RVD scores lower than 5%. This method has not been tested in patients and one can expect that the automatic contour optimization may suffer from fat infiltration as it is a gradient-based approach.

The propagation of an initial manual segmentation to the remaining slices through a combination of nonlinear registration approaches has been originally reported by Ogier et al. The method takes advantage of the shape information from the initial manual segmentations with no other previous information regarding muscles shapes. It is also based on the anatomical information from a given image to the next in order to take into account the corresponding changes along the proximo-distal axis. On that basis, the initial segmentation could be automatically propagated along this axis. The initial manual segmentation has to be repeated each time a muscle was appearing or disappearing. The method was initially proposed and validated for the segmentation of the four muscles of the *quadriceps femoris* group in T_1_-weighted images of 11 healthy subjects (58). Validation has been then extended for the segmentation of all individual thigh muscles in healthy subjects (59). Mean DSC scores of 0.90 ± 0.03 was reported with a manual input for 30% of the slices only. The semi-automatic method was also assessed for the segmentation of thigh and lower leg individual muscles in 10 patients with myotonic dystrophy type 1 (60). Using Dixon images recorded in both thigh and lower leg, only 9 out of 50 slices were manually segmented. Using water images, a mean DSC value of 0.91 ± 0.04 was reported and the results were similar regardless of the type of Dixon image used. In addition, an excellent reliability was also quantified from the comparative analysis between fat fraction computed from the segmented images and from the manual segmentation. The method has been deemed sufficiently robust for clinical applications and assayed in cross-sectional studies, which evaluated the pattern of fat infiltration in muscles in two different neuromuscular pathologies (12, 16).

Nonlinear registrations were also used for semi-automated segmentation methods dedicated to longitudinal studies.

Single-atlas approach was first assayed for the 3D segmentation of anterior and posterior thigh compartments in a 2-years follow-up study of patients with facioscapulohumeral muscular dystrophy, some at severe stages of intramuscular fat infiltration (61). No metrics of segmentation accuracy were reported but the fat quantification estimated through the semi-automatic segmentation showed a good reproducibility and repeatability as well as a good correlation with clinical scores.

Based on the single-atlas approach, Ogier et al. (60) reported a follow-up study in 10 myotonic dystrophy type 1 patients assessed twice 10 months apart. The 3D supervised segmentation of the first time point using the original semi-automatic method previously proposed (58) was used as a template for the automatic propagation to the second time point. The combined methods allowed an accurate segmentation with a DSC of 0.87 ± 0.07. Similarly to what has been obtained for the semi-automatic segmentation of baseline images, an excellent reliability was also observed between the fat fraction quantified from the automatic and manual segmentations. The combined methods provided the first complete framework dedicated to individual muscles segmentation and follow-up in patients with a neuromuscular disorder. Both the segmentation and a quantitative metric (fat fraction) in individual muscles were accurate, while the number of slices manually segmented was substantially reduced. The follow-up segmentation was performed with no additional manual segmentations and this could be translated for multiple repeated time points.

## 4. Deep Learning-Based Segmentation Methods

In the previous sections, evidence has been provided indicating that completely automatic methods are not efficient for a robust segmentation of individual fat-infiltrated muscles. On the contrary, semi-automatic methods can be robust and useable for clinical applications but the manual initialization remains an issue in terms of user dedication. Given the recent promising results reported in the field of medical imaging, deep learning-based segmentation methods are appealing (62).

Deep learning methods are part of machine learning methods, which have proven their efficiency in the diagnosis of neuromuscular diseases. Machine learning algorithm such as random forest models have been able to overcome experts for complicated diagnostic tasks (63). Deep learning methods are generally based on artificial neural networks, which are supervised to learn the segmentation process from manually segmented images provided as training examples. Neural networks rely on pixel intensities and image characteristics in order to compute the final segmentation. For images segmentation tasks, network architectures are built on the basis of convolutional encoder-decoder (CED) network. This kind of networks combines paired encoder and decoder networks and have the advantage of producing a result with a resolution similar to the initial images. This architecture can be seen as the association of a contracting path to capture the context and a symmetric expanding path that allows the image reconstruction. Various CED have been used for images segmentation. Among them, U-Net is considered as the standard CED architecture for image classification tasks (64) because of its efficient way of reconstructing the segmentation using information from the contracting path.

Similar to the conventional methods discussed in the previous sections, deep learning based segmentation methods have been applied for the different segmentation strategies, i.e., from the separation of muscle and fat deposits to the segmentation of individual muscles.

The very first deep learning approach applied on lower limb MR images was used in order to detect the *fascia lata*. Two studies intended to address this issue using a 5-layer network combined with a dual active contour model (65) or a U-Net architecture (66). Yao et al. used T_1_-weighted images while Amer et al. showed the interest of combining T_2_-weighted and PD images for their study. Both of them provided high-quality results with DSC values larger than 0.97 ± 0.02. Distinction between adipose and healthy muscle tissue was performed using the same networks and the corresponding DSC values were also high, i.e., 0.91 (66) and 0.94 ± 0.07 (65) for muscle detection. Recently, impressive DSC scores of 0.97 were obtained with an improved U-Net structure using residual connections and dense blocks (67). However, such a classification did not allow to distinguish perimuscular and intramuscular adipose tissue. Using a U-Net architecture, Gadermayr et al. intended to segment healthy and fat-infiltrated muscle all-together on T_1_-weighted images, allowing the distinction of intramuscular from perimuscular adipose tissue (68). Given the complexity of this task, corresponding DSC values were smaller (around 0.88 ± 0.05), illustrating a poorer segmentation quality.

With a similar purpose of distinguishing intramuscular and perimuscular fat, studies have been conducted with the aim of segmenting individual muscles. The AlexNet network was used by Ghosh et al. (69) to produce a principal component analysis of the segmentation, leading to poor results with average DSC of 0.85 ± 0.09. Standard deviation score illustrated the high variability of the results, which is known as a major weakness of deep learning methods (70). Better results were obtained with a U-Net architecture on T_1_-weighted images (71) with average DSC reaching 0.95 ± 0.03. More recently, Ding et al. (72) used U-Net on fat-water decomposition MR images to segment 4 muscle regions and obtained DSC scores around 0.89 ± 0.03 in both healthy and affected subjects. However, as the network was trained with a single slice position, the high muscle shape variability along the proximo-distal axis could not be taken into consideration. One way to take into account the variability of the proximo-distal shape is to consider the muscle as a volume, which can be done using a 3D segmentation neural network. In this field, Conze et al. (73) demonstrated the interest of 3D segmentation for the segmentation of individual shoulder muscles. A limitation to 3D CED is the memory necessary to train it. Ni et al. (74) used bounding boxes around organs to reduce resolution and prevent memory growth. This method was applied with 3D U-Net on complete lower limb images of athletes, obtaining mean DSC on 35 muscles of 0.89 ± 0.03. A very recent study proposed to use an edge-aware network based on U-Net and reached a DSC of 0.90 ± 0.09 and an ASD of 1.37 ± 0.92 on both healthy and affected subjects (75).

### Solutions to Scarcity of Data

The methods presented on the detection of *fascia lata*, the classification of adipose tissue, or the segmentation of individual muscles all showed promising results. However, all of them faced the problem of data availability due to the scarcity of annotated images of patients with pathological changes. Indeed, public datasets of annotated limbs MR images are scarce, unlike for other organs such as the brain (76).

An interesting solution for this issue relies on either the use of unlabeled data or the creation of artificial training examples. Amer et al. (66) proposed a semi-supervised learning method, which uses both labeled and unlabeled datasets. In that case, each image does not have to be annotated before the network training phase and one can increase the database without a human intervention for the labeling process. Anwar et al. (67) proposed to use a CED on unlabeled data to create labels and thus enlarge their dataset. However, unlabeled data are not always available especially for the study of rare diseases. For this purpose, methods based on data generation using neural networks are emerging since the founding article on generative adversarial networks (GAN) (77). Recently, Yi et al. (78) made a review regarding the application of such methods in medical imaging. For lower limb muscle segmentation, solutions based on GAN were assessed with the aim of generating pathological images (68). Many issues related to the realistic nature of the generated images and their variability have still to be addressed.

One has to keep in mind that the neural network training phase tightly relies on a tuning phase of the network hyper-parameters, which has to be empirically performed thereby reducing the fully automatic aspect of the method. In other words, a given network has to be optimized for a given dataset. Deep learning solutions have to be optimized by experts and this commonly takes hours of implementation. In addition, the training phases can be time consuming and the final results could be equivalent to those obtained with more conventional methods (53).

Overall, although deep learning tools for the segmentation of fat-infiltrated muscles have a great potential, one should keep in mind two major issues related to the availability of large amount of annotated data and the need of a specific network tuning for each dataset. Semi-automatic propagation methods have proven their efficiency (60) and could be used to annotate large amount of images. These methods could be combined to data augmentation for the generation of databases compatible with deep learning methods. The corresponding potential is still to be proven for the segmentation of fat-infiltrated muscles.

## 5. Conclusion

This review highlighted the lack of fully automated approaches that could produce accurate segmentations of muscle images of patients with neuromuscular disorders. The few validated methods that addressed the difficulty of segmenting images with severe infiltrated muscles were proposed for the whole muscle only. That might not be optimal in neuromuscular disorders in which individual muscles are seen to be affected differently. However, for segmentation of individual muscles, approaches that validated segmentations that were accurate enough for clinical use were evaluated only on healthy subjects. Specific studies are warranted for the extrapolation of these approaches to images of pathological muscles as the confounding factors differ. Indeed, the issue of distinguishing muscle, intramuscular adipose tissue, and subcutaneous adipose tissue seems to be crucial for the follow up of patients with a severe fat infiltration. Semi-automatic methods has proven some efficiency in clinical context. But even if they reduce the manual load required for the study of large cohorts, some manual interventions are still needed. As recent techniques, deep learning based approaches are promising but they need databases that are representative enough of typical neuromuscular disease images. The community should promote the emergence of common dedicated image databases.

## Author Contributions

AO, M-EB, and DB: guarantors of integrity of entire study. All authors: study concepts, manuscript drafting or manuscript revision for important intellectual content, approval of final version of submitted manuscript, agrees to ensure any questions related to the work are appropriately resolved, literature research, and manuscript editing.

## Conflict of Interest

The authors declare that the research was conducted in the absence of any commercial or financial relationships that could be construed as a potential conflict of interest.
